# A Cre-conditional *MYCN*-driven neuroblastoma mouse model as an improved tool for preclinical studies

**DOI:** 10.1038/onc.2014.269

**Published:** 2014-09-01

**Authors:** K Althoff, A Beckers, E Bell, M Nortmeyer, T Thor, A Sprüssel, S Lindner, K De Preter, A Florin, L C Heukamp, L Klein-Hitpass, K Astrahantseff, C Kumps, F Speleman, A Eggert, F Westermann, A Schramm, J H Schulte

**Affiliations:** 1 Department of Pediatric Oncology and Hematology, University Children's Hospital Essen, Essen, Germany; 2 German Cancer Consortium (DKTK), Partner Site Essen/Duesseldorf, Hufelandstr, Germany; 3 Center for Medical Genetics Ghent (CMGG), Ghent University Hospital, De Pintelaan 185, Ghent, Belgium; 4 German Cancer Research Center (DKFZ), Im Neuenheimer Feld 280, Heidelberg, Germany; 5 Translational Neuro-Oncology, West German Cancer Center, University Hospital Essen, University Duisburg-Essen, Essen, Germany; 6 Institute of Pathology, University Hospital Cologne, Cologne, Germany; 7 New Oncology -a division of Blackfield AG, Köln, Germany; 8 Institute of Cell Biology (Cancer Research), Faculty of Medicine, University of Duisburg-Essen, Essen, Germany; 9 Department of Pediatric Oncology, Hematology and BMT, Charité University Medicine, Augustenburger Platz 1, Berlin, Germany

## Abstract

Neuroblastoma, a childhood cancer that originates from neural crest-derived cells, is the most common deadly solid tumor of infancy. Amplification of the *MYCN* oncogene, which occurs in approximately 20–25% of human neuroblastomas, is the most prominent genetic marker of high-stage disease. The availability of valid preclinical *in vivo* models is a prerequisite to develop novel targeted therapies. We here report on the generation of transgenic mice with Cre-conditional induction of *MYCN* in dopamine β-hydroxylase-expressing cells, termed LSL-*MYCN*;Dbh-iCre. These mice develop neuroblastic tumors with an incidence of >75%, regardless of strain background. Molecular profiling of tumors revealed upregulation of the *MYCN*-dependent miR-17–92 cluster as well as expression of neuroblastoma marker genes, including tyrosine hydroxylase and the neural cell adhesion molecule 1. Gene set enrichment analyses demonstrated significant correlation with *MYC*-associated expression patterns. Array comparative genome hybridization showed that chromosomal aberrations in LSL-*MYCN*;Dbh-iCre tumors were syntenic to those observed in human neuroblastomas. Treatment of a cell line established from a tumor derived from a LSL-*MYCN*;Dbh-iCre mouse with JQ1 or MLN8237 reduced cell viability and demonstrated oncogene addiction to MYCN. Here we report establishment of the first Cre-conditional human *MYCN*-driven mouse model for neuroblastoma that closely recapitulates the human disease with respect to tumor localization, histology, marker expression and genomic make up. This mouse model is a valuable tool for further functional studies and to assess the effect of targeted therapies.

## Introduction

Neuroblastoma is the most common deadly solid tumor of infancy, and accounts for 15% of pediatric cancer deaths.^1,2^ Primary tumors derive from precursor cells of the sympathetic nervous system along the sympathetic chain.^3^ Neuroblastoma most frequently arises in the adrenal, but also develops from the superior cervical or celiac ganglia.^4^ The most prominent genetic marker of high-stage disease is amplification of the *MYCN* oncogene, which occurs in 20–25% of all neuroblastomas.^5^ Mouse models for human neuroblastoma can be helpful to understand the molecular mechanisms underlying pathogenesis and serve as important tools for preclinical studies. Weiss *et al.* has previously demonstrated that *MYCN* has the potential to drive murine neuroblastoma in a transgenic model, in which *MYCN* expression is driven by a rat tyrosine hydroxylase (*TH*) promoter.^6^ Transgenic mice expressing *MYCN* in the abdominal ganglia developed neuroblastoma. Although representing an excellent and broadly used tool, some limitations exist: (i) the transgene integration site and its genomic context is ill-defined, potentially resulting in less robust *MYCN* expression, (ii) tumors predominantly originate from abdominal ganglion structures, thus resembling only a subset of human neuroblastoma, (iii) no intrinsic option for *in vivo* tumor imaging is included and (iv) tumor incidence of heterozygous *TH-MYCN* mice is 70% in the 129 × 1/SvJ strain background, but only 5% in C57Bl6/N background, reducing the potential for combination with other cancer-relevant alleles. We aimed to overcome these limitations by developing a novel mouse model with targeted Cre-conditional *MYCN* expression in the neural crest.

## Results

### Characterization of LSL-*MYCN* mice

**Figure 1:**
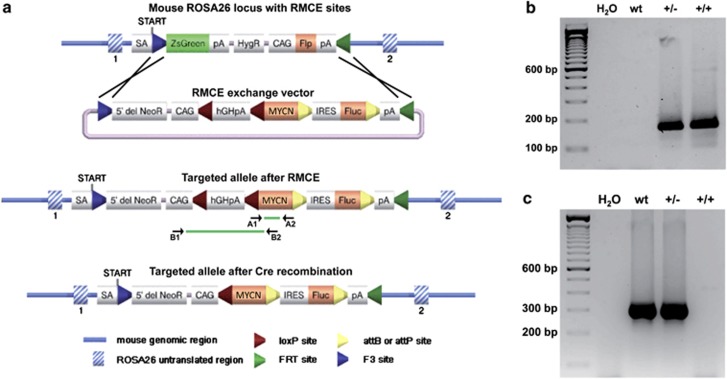
Generation of transgenic LSL-*MYCN* mice. (**a**) Graphical representation of the ROSA26 locus with recombinase-mediated cassette exchange (RMCE) sites used to introduce the RMCE exchange vector containing the *MYCN* transgene. The Rosa26 locus is displayed before (top) and after (center) insertion of *MYCN* by RMCE, and after cre-recombinase-mediated removal of the transcription termination site 5′ to the *MYCN* allele (bottom). Localizations of primers used for genotyping (A1 and A2) and the PCR-based validation of floxing out the transcriptional site 5′ of the *MYCN* allele (B1 and B2) are displayed. Splice acceptor site (SA), polyadenylation signal (pA), internal ribosome entry site (IRES), chicken actin gene promotor (CAG), transcriptional STOP cassette made of the human Growth Hormone polyadenylation signal (hGHpA), human *MYCN* open reading frame (MYCN). (**b**) Representative genotyping PCR validating the *MYCN* knock-in allele in heterozygous and homozygous LSL-*MYCN* mice (primers used: A1 and A2); wild type (wt), heterozygous LSL-*MYCN* (+/−), homozygous LSL-*MYCN* (+/+). (**c**) Representative PCR validating absence or presence of the transgene inserted into the *ROSA26* locus in wt, heterozygous (+/−) and homozygous (+/+) LSL-*MYCN* mice.

LSL-*MYCN* transgenic mice were viable and fertile without obvious physiological or morphological phenotypes. Offspring resulting from breeding LSL-*MYCN* mice with wild-type mice were born according to the expected Mendelian ratio (data not shown). We next confirmed the molecular integration site and localization of the transgene. Representative examples of PCR analyses validating transgene integration in heterozygous and homozygous LSL-*MYCN* mice are shown in Figure 1b. PCR analyses validating the presence of a wild-type allele at the ROSA26 locus in wild-type and heterozygous LSL-*MYCN* mice are shown in Figure 1c.

### Targeted expression of *MYCN* in the neural crest causes abdominal tumor formation in double-transgenic LSL-*MYCN*;Dbh-iCre mice

**Figure 2:**
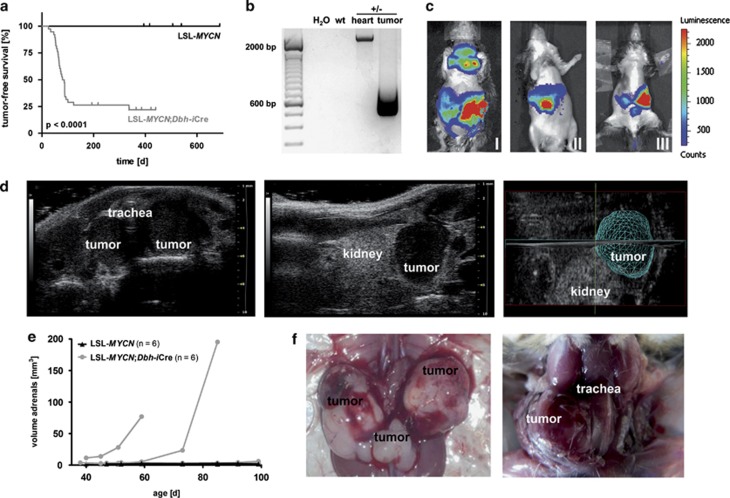
Double-transgenic LSL-*MYCN*;Dbh-iCre mice develop tumors derived from the neural crest. (**a**) Kaplan–Meier analysis indicating the presence and the time to detection of palpable tumors in mice (that is, tumor-free survival) heterozygous for LSL-MYCN and mice double transgenic for LSL-MYCN and Dbh-iCre (Log-rank test). (**b**) Representative result of PCR validating the removal or presence of the transcriptional termination site 5′ to the *MYCN* transgene in tumor and control tissues, respectively. Wild type (wt), double-transgenic LSL-*MYCN*;Dbh-iCre (+/−). (**c**) Bioluminescence imaging of three representative LSL-*MYCN*;Dbh-iCre mice carrying palpable tumors at the superior cervical ganglion (I), adrenals (I, II, III) or celiac ganglion (III). Color code indicates luciferase activity (low=blue; high=red). (**d**) High frequency ultrasound images of palpable tumors arising from superior cervical ganglion (left) and adrenal (middle), and three-dimensional reconstruction of adrenal tumor (right). (**e**) Growth curves of tumors, as detected by high-frequency ultrasound. (**f**) Macroscopic images during autopsy of mice carrying palpable tumors arising from both adrenals and the celiac ganglion (left) and from the superior cervical ganglion (right).

Double-transgenic LSL-*MYCN*;Dbh-iCre mice developed palpable abdominal tumors with an incidence of 76% in a mixed C57/Bl6/129 × 1/SvJ strain background (*n*=38). Tumor onset was detectable between 26–337 days of age, and the mean age at tumor onset was 79.6 days (Figure 2a). Interestingly, Kaplan–Meier analysis revealed a significantly prolonged tumor-free survival of mice heterozygous for *TH-MYCN* compared with LSL-*MYCN*;Dbh-iCre mice (*P*=0.001; Supplementary Figure 1). Of note, none of the single transgenic LSL-*MYCN* or Dbh-iCre mice developed a tumor (Figure 2a); *P*<0.0001. Cre-mediated recombination in tumors, but not in control tissue from LSL-*MYCN*;Dbh-iCre mice was validated using B1 and B2 primers (Figure 1a). In the presence of the transcriptional termination site, a 2241-bp band was present in control tissue, whereas Cre-mediated excision of the transcriptional termination site upstream of the *MYCN* allele was indicated by the presence of a 703-bp band (Figure 2b and Supplementary Figure 2).

### Tumor localization, histology and expression of marker genes in LSL-*MYCN*;Dbh-iCre mice recapitulate the patterns of human neuroblastoma

**Figure 3:**
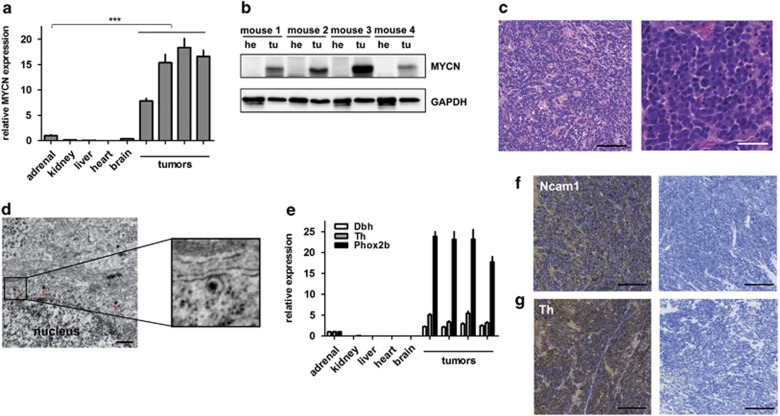
Tumors of LSL-MYCN;Dbh-iCre mice resemble human neuroblastoma in terms of histology and molecular expression patterns. (**a**) *MYCN* expression (qPCR) in four representative tumors from LSL-*MYCN*;Dbh-iCre mice compared with control tissues. Expression was normalized to normal adrenal glands. Student's *t*-test: ***P<0.001. (**b**) Western blot analysis confirms MYCN expression in tumors (tu) compared with heart (he) tissue collected from four representative double-transgenic mice. (**c**) Hematoxylin and eosin (H&E) staining shows small, round blue cells typical for neuroectodermal tumors. Scale bars=100 μm (left) and 50 μm (right). (**d**) Electron micrographs show neuronal structures, including neurosecretory vesicles (red arrows). Scale bar=500 nm. (**e**) Reverse transcription-qPCR confirms significantly increased expression of the murine orthologs of the human neuroblastoma marker genes dopamine β-hydroxylase (*Dbh*), tyrosine hydroxylase (*Th*) and paired-like homeobox 2b (*Phox2b*) in tumors compared with normal control tissues (Student's *t*-test; *Dbh*: *P*=0.005, *Th*: *P*=0.02, *Phox2b*: *P*=0.0006). (**f**, **g**) Immunohistochemistry confirms expression of neuroblastoma markers, Ncam1 and Th. Scale bars=200 μm.

*In vivo* bioluminescence imaging (Figure 2c) revealed that tumors in LSL-*MYCN*;Dbh-iCre mice arose from the superior cervical ganglion (I), the adrenals (I, II, III) or the celiac ganglion (III). Several tumors were shown to originate from adrenal structures using high-frequency ultrasound imaging (Figures 2d and e). Macroscopic images acquired during dissection of mice carrying palpable tumors (Figure 2f) also confirmed that tumors arose from both adrenals and the celiac ganglion (left) and from the superior cervical ganglion (right). Tumors had elevated expression of both *MYCN* mRNA and protein compared with normal tissues (Figures 3a and b). Hematoxylin and eosin staining of histological tumor sections and electron microscopy showed a small round blue cell tumor with cells harboring neurosecretory vesicles (Figures 3c and d), indicative of neuroblastoma. Furthermore, tumors strongly expressed the neuroblastoma-specific marker genes, dopamine β-hydroxylase (*Dbh*), tyrosine hydroxylase (*Th*) and paired-like homeobox 2b (*Phox2b*), as observed by quantitative PCR (qPCR; Figure 3e). The neural cell adhesion molecule, Ncam1, a marker for neuroendocrine tissues, was strongly expressed in tumors from LSL-*MYCN*;Dbh-iCre mice (Figure 3f). Expression of tyrosine hydroxylase was also confirmed on the protein level (Figure 3g). To analyze early or even premalignant stages of neuroblastoma development in our mouse model, we collected adrenals for histological examinations at day of birth, at days 14 and 28 of life (Supplementary Figures 3a–c). Hyperplastic cells were present in the adrenal medulla of some, but not all, of adrenal glands from 0-day-old transgenic mice and most 14- and 28-day-old transgenic mice. Especially at day 28, the adrenal medulla of LSL-*MYCN*;Dbh-iCre mice had an atypical, nodal tissue architecture. In contrast, no hyperplasia was observed in adrenal glands from control mice of any age. The observed hyperplasia is in line with the hyperplastic lesions (in superior cervical ganglia) previously described in TH-*MYCN* mice by Hansford *et al.*^7^ Taken together, we demonstrate that LSL-*MYCN*;Dbh-iCre mice develop neuroblastomas that arise from the adrenal medulla and other neural crest derivatives.

### Murine *MYCN*-driven neuroblastomas are characterized by genomic aberrations syntenic to human neuroblastomas

**Figure 4:**
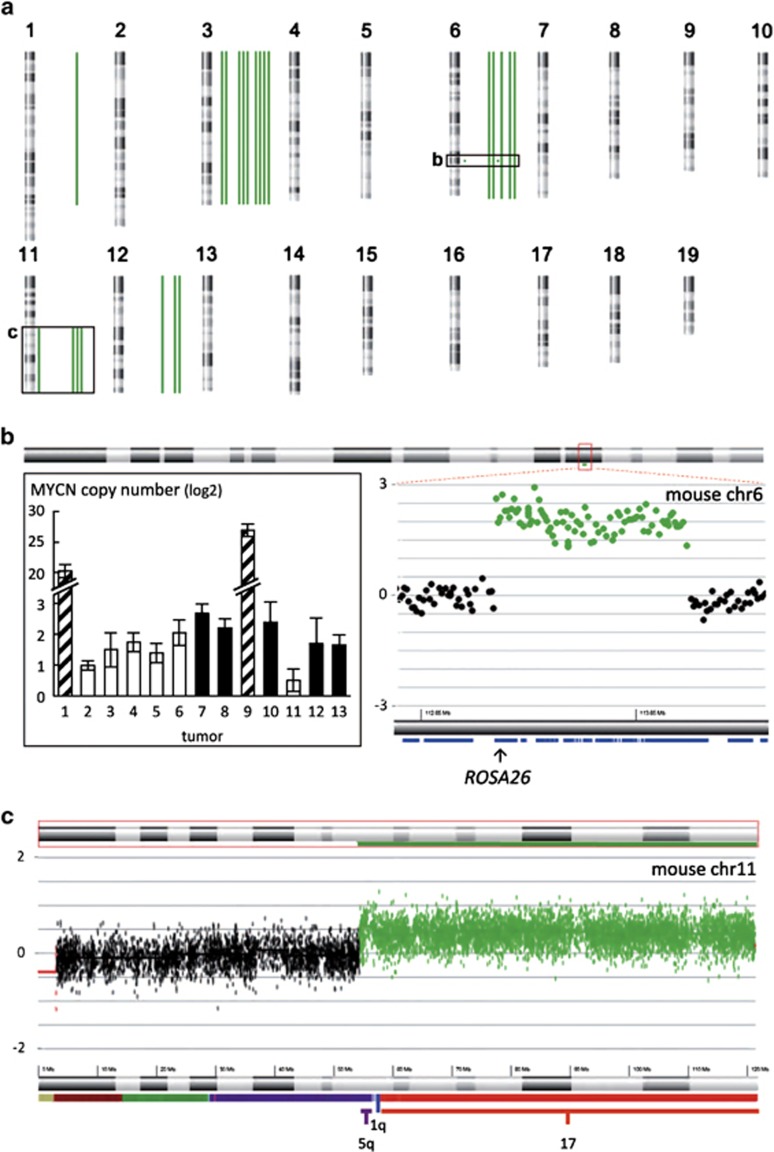
Murine neuroblastomas recapitulate genomic aberrations of human neuroblastomas. (**a**) Mouse karyotype overview of all genomic imbalances detected in 13 murine neuroblastomas (green bars: gained regions, red bars: lost regions). (**b**) Partial ratio plot for the mouse chromosome 6 region encompassing the *ROSA26* amplicon in tumor 9 (right) and copy number of the *MYCN* transgene in 13 tumors from heterozygous LSL-*MYCN*;Dbh-iCre mice (+/−), as assessed by qPCR (insert left). Bars represent mice with normal chromosome 6 copy number (white), with whole chromosome 6 gain (black) and with focal chromosome 6 amplification (striped). (**c**) Tumor/control ratio plot for mouse chromosome 11 in tumor 9 showing partial chromosome 11q gain, corresponding to gain of almost the entire human chromosome 17.

An overview of all genomic aberrations detected in tumors from heterozygous LSL-*MYCN*;Dbh-iCre mice compared with tail DNA is depicted in Table 1 and Figure 4a. A partial gain of murine chromosome 11q was detected in the tumors of four mice (Figures 4a and c). This region is syntenic to human chromosome 17 (Figure 4c), for which gain or partial gain (17q) occurs in the majority of human neuroblastomas.^8^ Five tumors exhibited gain of the entire murine chromosome 6 (Figure 4a and Supplementary Figure 4c), which is partially syntenic to human chromosomes 7p, 7q and 12p. In human neuroblastomas, gain of an entire chromosome 7 occurs in 40% of tumors and appears to be prevalent in all tumor stages, whereas gain of 7q, observed in 12% of human tumors, is more common in higher stage tumors.^9^ Three tumors harbored a gain of the entire mouse chromosome 12, which is syntenic to human chromosome 2p and includes the *MYCN* locus, as well as human chromosome 14q and parts of human chromosome 7p and 7q (Figure 4a and Supplementary Figure 4d). Eight tumors displayed gain of the entire chromosome 3 (Figure 4a and Supplementary Figure 4b), which is partially syntenic to human chromosome 1q, a region often gained in human neuroblastomas.^10^ Interestingly, two tumors showed a focal gain on chromosome 6 that encompassed the *ROSA26* locus, in which the human *MYCN* transgene was integrated (Figures 4a and b). This focal gain resulted in a 20- to 25-fold increase in *MYCN* transgene copy number in these two tumors, as measured by qPCR (inset Figure 4b). Consequently, the two tumors containing this aberration had elevated expression of the human *MYCN* mRNA (Supplementary Figure 4e), as assessed by reverse transcription-qPCR.

No chromosomal deletions and only few focal deletions were observed in the tumors from heterozygous LSL-*MYCN;Dbh-iCre* mice. The region on murine chromosome 8 that was lost in two murine tumors, which is syntenic to human chromosome 4, does not harbor any annotated genes (data not shown). Each of the remaining focal losses were only observed in one tumor, and are not syntenic to regions often lost in human neuroblastomas. From these data, it appears that chromosomal and focal losses observed in human neuroblastomas are less well represented in the LSL-*MYCN;Dbh-iCre* mouse model. Nevertheless, the spectrum of chromosomal aberrations in these *MYCN*-driven murine tumors recapitulates many of the observed chromosomal imbalances observed in human neuroblastomas.

### Murine *MYCN*-driven neuroblastoma transcriptomes show patterns of canonical MYC-related mRNA and microRNA (miRNA) signatures

**Figure 5:**
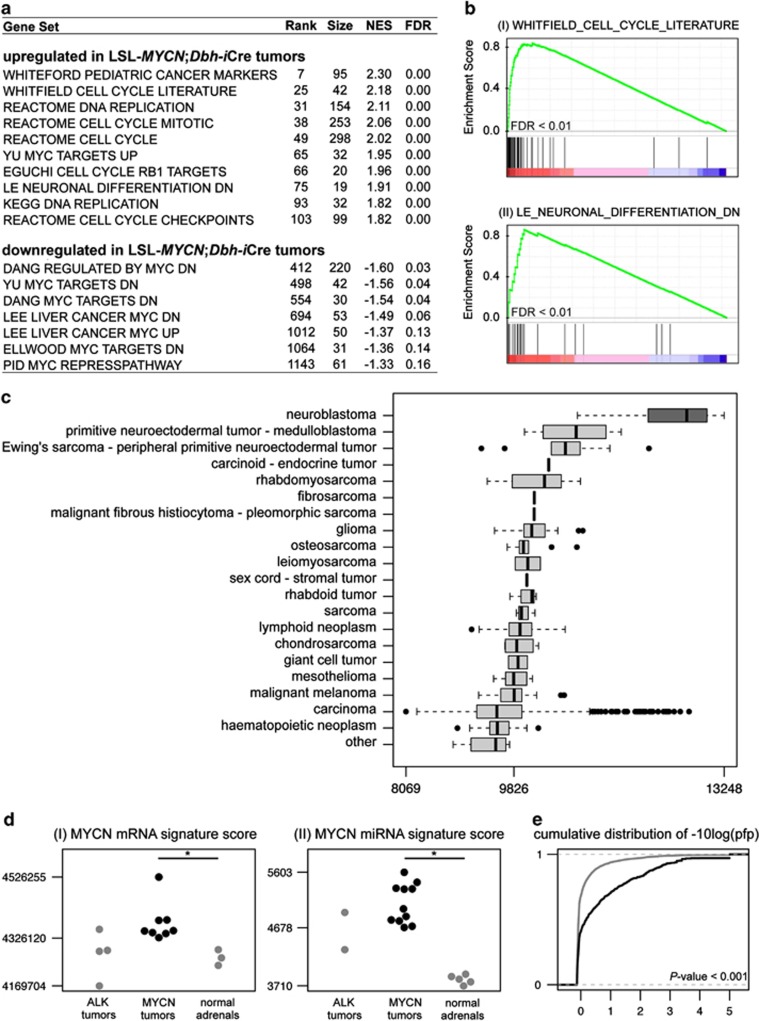
Tumors from heterozygous LSL-MYCN;Dbh-iCre mice recapitulate human neuroblastoma at transcriptional level. (**a**) Table of selected gene sets from the MSigDB C2 collection, enriched among genes upregulated in the tumors from heterozygous LSL-*MYCN*;Dbh-iCre mice based on GSEA. (Rank of gene set in overall list of gene sets, ranked according to decreasing normalized enrichment score (NES; rank), number of genes in each set (size), NES). (**b**) GSEA enrichment plots showing upregulation of a gene set representing cell cycle (I) and markers downregulated during neuronal differentiation (II) in the transcriptional profiles of neuroblastoma tumors from heterozygous LSL-*MYCN*;Dbh-iCre mice. Depicted is the plot of the running sum for the MSigDB gene set within the LSL-*MYCN*;Dbh-iCre neuroblastoma data set, including the maximum enrichment score and the leading edge subset of enriched genes. FDR = false discovery rate. (**c**) The LSL-*MYCN*;Dbh-iCre signature score in the 967 cell lines in the Cancer Cell Line Encyclopedia^13^ showing the highest signature score in neuroblastoma cell lines, followed by medulloblastoma cell lines. (**d**) The MYCN mRNA (I) and miRNA (II) gene signature in normal adrenal medulla and MYCN- and *ALK*^*F1174L*^-driven tumors. *P*<0.05 (*) was considered significant. (**e**) The cumulative distribution of the significance score [-10log(pfp)] associated with differential expression in tumors from heterozygous LSL-*MYCN*;Dbh-iCre mice (+/−) versus normal adrenals, for genes in the human non-*MYCN*-amplified neuroblastoma signature (black) and all other genes (gray). Genes in the human non-*MYCN*-amplified neuroblastoma signature show more significant differential expression compared with all remaining genes (Kolmogorov–Shmirnov test, *P*<0.001).

The consequences of *MYCN* overexpression on downstream gene expression were analyzed using transcriptional profiles obtained from normal murine adrenal gland and tumors from LSL-*MYCN*;Dbh-iCre mice. Unsupervised hierarchical clustering using the 1% of genes with the highest standard deviation in expression across all samples revealed distinct clustering of normal adrenal medulla and *MYCN*-driven tumors (Supplementary Figure 5). Interestingly, a similar hierarchical clustering approach revealed that tumors from LSL-*MYCN*;Dbh-iCre mice are very similar to tumors from the well-established TH-*MYCN* mouse model,^6^ both at the level of mRNA and miRNA expression (Supplementary Figures 6a and b). Consistent with the role of *MYCN* as a transcriptional activator, there was a predominance of upregulated over downregulated genes: 2315 genes, represented by 3680 probe sets, were significantly upregulated, whereas 1190 genes, represented by 1726 probe sets, were significantly downregulated (false discovery rate <0.05; Supplementary Table 2). The differential expression profiles of regulated transcripts were functionally annotated using Gene Set Enrichment Analysis (GSEA),^11^ which seeks to estimate the significance of overrepresentation of an independently defined set of genes in gene expression data sets. LSL-*MYCN*;Dbh-iCre tumors were characterized by a strong association with MYC-upregulated target genes, whereas MYC-downregulated target genes were enriched in the normal adrenal glands (Figure 5a). In addition, a large number of DNA replication-related gene sets were statistically enriched among genes upregulated in LSL-*MYCN*;Dbh-iCre tumors (Figures 5a and b(I)). Furthermore, upregulation of a gene set representing genes repressed during neuronal differentiation is in line with the undifferentiated phenotype of LSL-*MYCN*;Dbh-iCre tumors (Figures 5a and b(II)). Finally, one of the most strongly enriched gene sets among genes upregulated in the *MYCN*-driven tumors is the WHITEFORD_PEDIATRIC_ CANCER_MARKERS gene set, consisting of differentially expressed genes in a panel of xenografts representing eight common pediatric tumors (neuroblastoma, rhabdomyosarcoma, Ewing sarcoma, acute lymphoblastic leukemia, Wilms' tumor, osteosarcoma, medulloblastoma and ependymona), compared with normal tissues^12^ (Figure 5a). This pediatric cancer phenotype is also apparent when comparing a LSL-*MYCN*;Dbh-iCre signature in 967 cancer cell lines from Cancer Cell Line Encyclopedia,^13^ representing more than 20 tumor entities. The LSL-*MYCN*;Dbh-iCre signature, composed of the top 100 most differentially up- and downregulated genes in LSL-*MYCN*;Dbh-iCre tumors compared with normal adrenal gland, is significantly higher in neuroblastoma cell lines than in any other cancer cell line (Kruskal–Wallis rank sum test, *P*<0.001; Figure 5c). To specifically evaluate the magnitude of *MYCN* activity on transcriptional profiles both at mRNA and miRNA level, a *MYCN* signature score^14^ was calculated for all samples. As an additional reference, profiles from neuroblastoma tumors arising from targeted overexpression of mutated *ALK*^15^ were included. The *MYCN* signature score was significantly higher in *MYCN*-driven tumors than in the *ALK*^*F1174L*^-driven tumors and normal murine adrenals, demonstrating that *MYCN* is strongly activated in the tumors arising in transgenic mice with targeted *MYCN* expression (Figures 5d(I), Supplementary Figure 7a and Supplementary Table 3). Known human MYCN-upregulated (*Cad*, *Cdk4*, *Odc1*) and MYCN-downregulated (*Dkk3*, *Rgs5*) target genes were also significantly regulated in LSL-*MYCN*;Dbh-iCre tumors compared with normal adrenals from wild-type mice (Supplementary Figure 7b). The miRNA expression profiles obtained from normal murine adrenal gland and both MYCN- and *ALK*^*F1174L*^-driven neuroblastomas showed similar *MYCN*-driven patterns (Figure 5d (II), Supplementary Figure 8 and Supplementary Table 4). Of the 380 expressed miRNAs that were measured on the platform, 26 miRNAs were upregulated and 38 miRNAs were downregulated in LSL-*MYCN;Dbh-iCre* tumors compared with normal murine adrenal gland (Supplementary Table 5). Several miRNAs from the MYCN-induced miR-17–92 cluster (miR-17-5p, miR-18-5p, miR-20a-5p and miR-92a-3p) were significantly upregulated in LSL-*MYCN*;Dbh-iCre tumors compared with normal adrenals from wild-type mice (Supplementary Figure 8b). The conformity with human neuroblastoma is further supported by the observation that genes, differentially expressed between high- and low-risk human neuroblastomas, either without or with inclusion of *MYCN*-amplified tumors, are more significantly altered in LSL-*MYCN*;Dbh-iCre tumors compared with normal adrenals from wild-type mice (Kolmogorov–Shmirnov test, *P*<0.001; Figure 5e). In summary, these observations support the relevance of our new *MYCN*-driven mouse model for the study of human neuroblastoma.

### The mNB-A1 cell line, explanted from LSL-*MYCN*;Dbh-iCre tumors, reflects characteristics of its origin

**Figure 6:**
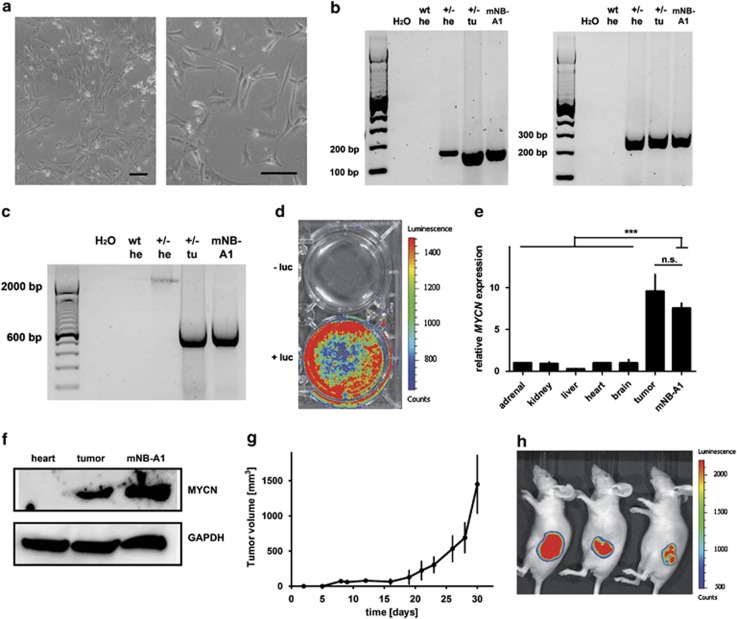
(**a**) Macroscopic images of cells cultivated after explantation of LSL-*MYCN*;Dbh-iCre tumors. Scale bars=100 μm. (**b**) Representative genotyping PCR validating the *MYCN* knock-in allele (left) and the *Dbh-iCre* transgene (right) in cells cultured from murine neuroblastoma. Heart (he) from wild-type (wt) and heart (he) and tumor (tu) from heterozygous LSL-*MYCN;*Dbh-iCre mice (+/-) as controls. (**c**) PCR validating the removal of the transcriptional termination site 5′ of the *MYCN* allele in cells cultivated after explantation of LSL-*MYCN*;Dbh-iCre tumors. Wild-type (wt), heterozygous LSL-*MYCN*;Dbh-iCre (+/−), heart (he), tumor (tu). (**d**) Bioluminescence imaging of mNB-A1 cells. Luciferase activity: low=blue; high=red. luciferin (luc). (**e**) *MYCN* expression (qPCR) in mNB-A1 cells compared with various control tissues and to a representative LSL-*MYCN*;Dbh-iCre tumor. Expression was normalized to that in normal adrenal glands. Student's *t*-test: ***=*P*<0.001; NS=not significant. (**f**) Western blot analysis confirms MYCN expression in mNB-A1 cells compared with heart and LSL-*MYCN*;Dbh-iCre tumor. (**g**) Tumor growth after engraftment of 10^7^ mNB-A1 cells into three nude mice at day 0. (**h**) Bioluminescence imaging of mNB-A1 cells growing in nude mice. Luciferase activity: low=blue, high=red.

*In vitro* cultured cells derived from a tumor of a LSL-*MYCN*;Dbh-iCre mouse presented neuronal structures resembling human neuroblastoma cells (Figure 6a). The presence of both MYCN and the Dbh-iCre transgene as well as Cre-mediated recombination in tumor-derived mNB-A1 cells was validated (Figures 6b and c). Furthermore, mNB-A1 cells were positive for luciferase expression as revealed by bioluminescence imaging (Figure 6d) and expressed MYCN mRNA and protein levels similar to LSL-*MYCN*;Dbh-iCre tumors (Figures 6e and f). To monitor their tumorigenic potential *in vivo*, mNB-A1 cells were inoculated into nude mice (Figure 6g). Bioluminescence imaging revealed that tumors from mNB-A1 cells maintained their luciferase activity (Figure 6h), and that luciferase imaging could be used to follow these tumors *in vivo*. Analysis of re-grafted tumors in nude mice revealed a strong correlation of tumor size with activity detected by luciferase imaging (Supplementary Figure 9).

**Figure 7:**
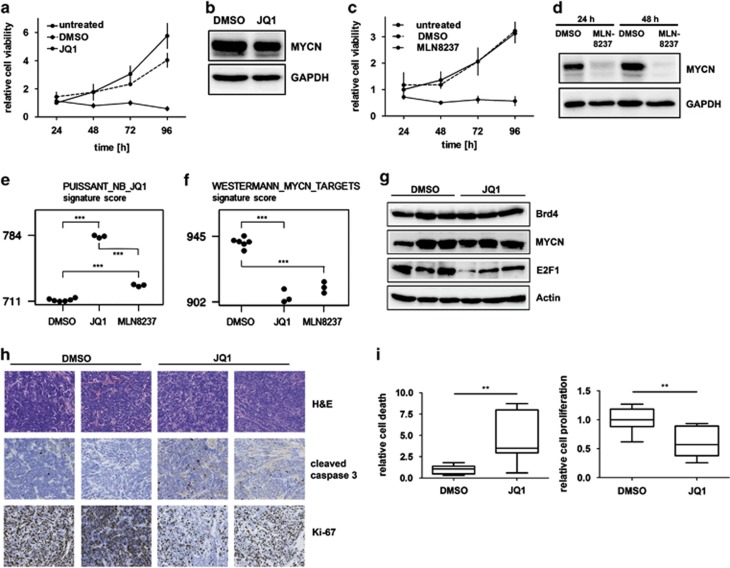
Treatment of mNB-A1 and re-grafted tumors. (**a**) JQ1 treatment of mNB-A1 cells significantly reduced cell viability in MTT assays compared with untreated or DMSO-treated cells. (**b**) Western blot analysis confirmed no MYCN protein regulation in mNB-A1 cells treated with JQ1 compared with DMSO-treated cells. (**c**) MLN8237 treatment of mNB-A1 cells significantly reduced cell viability in MTT assays compared with untreated or DMSO-treated cells. (**d**) Western blot analysis confirmed MYCN downregulation in mNB-A1 cells treated with MLN8237 compared with DMSO-treated cells. (**e, f**) A mRNA signature score established from genes differentially expressed after JQ1 treatment of neuroblastoma cell lines^16^ (e), and a MYCN mRNA signature score^14^ (**f**) for mNB-A1 cells treated with either DMSO, JQ1 or MLN8237. (**g**) Western blot analyses of Brd4, MYCN, Myc, E2f1 and Cyclin D1 expression in re-grafted tumors from LSL-*MYCN*;Dbh-iCre mice treated with JQ1 or DMSO. Actin and Gapdh were used as loading controls. (**h**) Re-grafted tumors from JQ1- or DMSO-treated mice were examined histologically after hematoxylin/eosin (H&E) staining or immunostaining for cleaved caspase 3 (apoptotic cells) or Ki-67 (actively proliferating cells). Representative images are shown. (**i**) Bar graphs show the mean relative apoptosis (left) and proliferation (right) calculated from three representative images from each re-grafted tumor from groups of mice treated with either JQ1 or DMSO. Significance was calculated by Student's *t*-test: *P<0.05, **P<0.01, ***P<0.001.

Treatment of cultured mNB-A1 cells with JQ1, a pharmacological inhibitor with a high target potency against BET bromodomain proteins,^16^ significantly reduced cell viability (Figure 7a). Although MYCN expression in mNB-A1 cells remained unaltered by treatment with either JQ1 or the dimethyl sulfoxide (DMSO) control (Figure 7b), transcriptomic data closely mimic the observed transcriptional changes after JQ1 treatment of neuroblastoma cell lines^16^ (Figure 7e). Furthermore, a strong reduction in MYCN signatures was observed, confirming the effect of JQ1 treatment on MYCN transcriptional programs (Figure 7f and Supplementary figure 10). Treatment with MLN8237, a pharmacological inhibitor that decreases MYCN protein levels by abolishing autophosphorylation of Aurora A,^17^ also significantly reduced cell viability (Figure 7c) and MYCN expression (Figure 7d). Gene expression profiles of mNB-A1 cells after MLN8237 treatment show similarities with the transcriptional changes observed after JQ1 treatment of human neuroblastoma cell lines^16^ (Figure 7e), and closer similarities to mNB-A1 cells treated with JQ1 (Figure 7f and Supplementary Figure 10). These data support a MYCN transcriptional program repression resulting from both MLN8237 or JQ1 treatment, although slight differences are observed, which is to be expected for treatment with compounds having a different mode of action. We conclude that cells derived from tumors of LSL-*MYCN*;Dbh-iCre mice retained their malignant capacity upon transplantation, and that growth remains depended on *MYCN* function, indicating oncogene addiction.

### JQ1 treatment induces apoptosis and decreases proliferation of re-grafted tumors from LSL-*MYCN*;Dbh-iCre tumors

We next aimed to analyze whether JQ1 treatment also affects tumors from LSL-*MYCN*;Dbh-iCre mice *in vivo*. For that purpose, nude mice harboring re-grafted tumors from LSL-*MYCN*;Dbh-iCre mice were treated with JQ1. Western blotting of fresh-frozen re-grafted tumor lysates showed that JQ1 treatment did not suppress MYCN or Brd4 protein expression in the tumors (Figure 7g), but clearly downregulated E2f1 protein expression. Immunhistochemical analysis of the tumors revealed that JQ1 treatment also significantly decreased Mib-1 (Ki-67) expression, as an indicator of cell proliferation, and increased the level of cleaved caspase 3, indicating the induction of apoptosis (Figures 7h and i). Taken together, the effects of JQ1 treatment *in vitro* were recapitulated *in vivo*.

## Discussion

Although treatment advances in many pediatric cancer types have resulted in increased survival of affected patients, the prognosis for advanced stage neuroblastoma remains poor, especially after tumor relapse.^18^ Preclinical models to better understand the molecular features of aggressive neuroblastoma and that can be used to evaluate novel therapies are urgently needed. Here, we present a novel conditional *MYCN*-driven mouse model that resembles human neuroblastoma. Using Cre expression driven by the *Dbh* promotor, which is active specifically in noradrenergic neurons of the peripheral and central nervous system,^19^ we restricted transgenic *MYCN* expression to the presumed tissue of neuroblastoma origin. Double-transgenic LSL-*MYCN*;Dbh-iCre mice developed tumors with a high incidence and regardless of strain background, thus overcoming one of the major limitations of existing models. Bioluminescence imaging identified tumors recapitulating human neuroblastoma localization and histology. LSL-*MYCN*;Dbh-iCre tumors also mimicked molecular marker expression and reflected chromosomal aberrations of human neuroblastomas. Interestingly, the most common genomic aberrations in human neuroblastomas, including gain of chromosome 17q, were also observed in tumors from LSL-*MYCN*;Dbh-iCre transgenic mice. Therefore, this mouse model offers the possibility for cross-species genomic analyses toward identifying the presumed oncogenic drivers on human chromosome 17q. In addition to a gain of the region of mouse chromosome 11, which is syntenic to human chromosome 17q, several other recurrent aberrations were observed that resemble aberrations observed in human neuroblastomas. Interestingly, chromosomal gains of human neuroblastomas are better recapitulated in our model than respective deletions. Specifically, loss of 1p36, a region frequently deleted in human *MYCN*-amplified neuroblastomas, was not recapitulated in our model. Our findings are in line with previous findings in the TH-*MYCN* neuroblastoma mouse model.^20,21^ Of note, numerical or partial gain of mouse chromosome 11 resembling human chromosome 17q gain and amplification of the *MYCN* transgene was observed in both *MYCN*-driven mouse neuroblastoma models. In addition, gain of mouse chromosome 3 has been observed in all analyzed neuroblastoma mouse models to date.^15,20, 21, 22^ Taken together, LSL-*MYCN*;Dbh-iCre mice develop neuroblastic tumors that share histological features and genomic alterations of human neuroblastomas.

Compared with the existing *MYCN*-driven mouse model of neuroblastoma, which expresses a human *MYCN* cDNA under the control of a rat tyrosine hydroxylase promoter,^6^ the novel LSL-*MYCN*;Dbh-iCre bears several advantages. First, transgene integration is better defined in LSL-*MYCN*;Dbh-iCre mice. The *ROSA26* locus is commonly used for the generation of genetically engineered knock-in mice, because it is ubiquitously expressed and a discontinuation of that locus causes no known phenotypic effect in mice.^23,24^ In contrast, it is not known whether transgene integration into the distal region of chromosome 18, reported for the *TH-MYCN* mice, also causes positional effects that could possibly have an impact on tumorigenesis.^21^ Second, tumors developing in *TH-MYCN* mice are limited to abdominal ganglion structures,^6^ whereas LSL-*MYCN*;Dbh-iCre tumors develop predominantly from both adrenals, but also develop from the celiac and superior cervical ganglia, thus covering all locations in which human neuroblastomas arise. Third, *TH-MYCN* tumor penetrance is only high in a genetically near pure 129 × 1/SvJ strain background, probably due to differential expression of specific modifiers.^25^ This hampers the combination with other transgenic mouse strains modifying neuroblastomagenesis, as they need to be backcrossed genetically to achieve the same tumor incidence. By contrast, the LSL-*MYCN*;Dbh-iCre model also develops tumors in other mouse strain backgrounds, such as C57Bl/6N, at a high frequency. Taken together, the novel LSL-*MYCN*;Dbh-iCre neuroblastoma mouse model overcomes the limitations of the only existing *MYCN*-driven neuroblastoma model, *TH-MYCN*.

Beyond the advantages discussed above, the new LSL-*MYCN*;Dbh-iCre is strikingly similar to the widely used TH-*MYCN* mouse model in regard to the occurrence of chromosomal alterations, mRNA and miRNA expression profiles. Therefore, subsequent experiments with the LSL-*MYCN*;Dbh-iCre mouse model can easily build on the many excellent results obtained with the TH-*MYCN* mouse model in the previous years. The LSL-*MYCN*;Dbh-iCre model also represents an excellent tool to evaluate new targeted therapies *in vivo*. However, the LSL-*MYCN*;Dbh-iCre mouse model can only be used to analyze those factors or compounds that regulate or interfere with the MYCN protein itself or MYCN downstream signaling. Regulators of *MYCN* transcription or interactors with the *MYCN* mRNA, such as miRNAs targeting the *MYCN* 3′-untranslated region, cannot be analyzed in this model, as the *MYCN* cDNA that is ectopically expressed lacks any regulatory untranslated regions. A mouse model in which neuroblastomas are driven by overexpressed endogenous *Mycn*, such as the LSL-*Lin28b*;Dbh-iCre mouse model,^22^ is more appropriate for the latter analyses.

We have used the LSL-*MYCN*;Dbh-iCre mouse cell line to evaluate two drugs, MLN8327 and JQ1, which are known to target MYCN. MLN8327 destabilizes the MYCN oncoprotein by inhibiting the interaction between MYCN and AURKA. We confirmed that MLN8327 treatment downregulates MYCN protein in our model cell line, leading to a strong reduction in cell viability and a decrease in the MYCN activity score. First, this confirms that cells derived from tumors arising in the LSL-*MYCN*;Dbh-iCre mice exhibit oncogene addiction to MYCN. Second, our results support and extend the preclinical evidence indicating MLN8327 as a promising targeted therapeutic agent to treat *MYCN*-amplified neuroblastoma. The mode of JQ1 action includes the downregulation of *MYC(N)* mRNA transcription and, thereby, MYCN protein expression. As MYCN is ectopically expressed in our model system, a downregulation of MYCN expression after BRD4 inhibition is not necessarily expected. In line with this, we observed no change in MYCN expression after JQ1 treatment. Alternative mechanisms that could explain why JQ1 treatment still reduces cell viability in our model system include either MYCN-independent effects of JQ1 or the interference of JQ1 with MYCN-driven transcription rather than with transcription of the *MYCN* gene itself. The significant decrease of the MYCN activity score, which we observed following JQ1 treatment, implicates the latter effect, at least in combination with MYCN-independent effects of JQ1. *P*-TEFb has been previously demonstrated to be a factor required for MYC-driven transcription.^26,27^ Therefore, *P*-TEFb is most likely also required for MYCN-driven transcription as well. As BRD4 is an important factor for *P*-TEFb recruitment,^28^ we hypothesize that inhibiting Brd4 by JQ1 impairs recruitment of *P*-TEFb, and thereby attenuates MYC(N)-driven transcription. Although not in the focus and beyond the scope of this manuscript, this hypothesis should be taken into account and explored where it has weight for explaining JQ1 treatment results in future experiments attempting to finely assess the mode of action and side effects of JQ1. These should also help delineate the mechanism by which JQ1 decreases the MYCN signature score without downregulating MYCN protein levels.

A prerequisite for using LSL-*MYCN*;Dbh-iCre mice in preclinical research is assessing the dynamics of tumor development and a sufficient treatment window. With the dynamics of tumor development that we observed in LSL-*MYCN*;Dbh-iCre mice, the acceleration or delay of tumor development as well as an increase or decrease in tumor incidence could be used as measurable end points of experimental manipulation, including the introduction of further genetic alterations. A treatment window must be defined to use the LSL-*MYCN*;Dbh-iCre mice for preclinical analysis of potential therapeutic compounds or therapeutic strategies. The treatment window is the time from tumor detection to the time the mouse succumbs to disease or must be killed due to tumor burden. Our ultrasonography experiments clearly indicate the presence of a sufficient treatment window in the LSL-*MYCN*;Dbh-iCre mouse model (Figure 2e), although the number of mice analyzed was too low to exactly define the boundaries of the treatment window. Exact delineation of the treatment window for the same animal model will also vary between studies, as it depends on the techniques used to detect and follow the tumor (palpation, luciferase, ultrasonography or nuclear resonance imaging), the end points used in the study (hyperplasia versus an established tumor) and the time at which a mouse must be killed due to tumor burden (ethical considerations and varying national animal protection regulations).

As genetic features of human neuroblastomas are preserved in LSL-*MYCN*;Dbh-iCre-induced tumors, positional approaches might help to identify other crucial driver genes in neuroblastoma development. Thus, a plethora of options is already available to uncover the full potential of LSL-*MYCN*;Dbh-iCre transgenic mice in terms of neuroblastoma genetics and future therapeutics, and we expect the LSL-*MYCN*;Dbh-iCre mouse model to be a valuable tool for the neuroblastoma research community. In fact, the LSL-*MYCN* mouse line has already been transferred to several laboratories, and is available on request to the research community. To prepare for a future expansion of requests, sperm and frozen embryos are being conserved to allow easier transfer, and the mouse line will be submitted to one of the available public repositories, such as the European Mouse Mutant Archive. As *MYC(N)* is a key driver of tumorigenesis not only in neuroblastoma, combination the conditional LSL-*MYCN* mouse with other cre-transgenic models bear the potential to also model tumorigenesis of other human malignancies.

## Materials and methods

### Generation of LSL-*MYCN* mice and tumor detection

Human *MYCN* (Ensembl gene ID: ENSG00000134323) was cloned downstream of a chicken actin gene (*CAG*) promoter followed by *loxP*-flanked strong transcriptional termination site (LSL). The transgene was placed upstream of an internal ribosome entry site (IRES) and a second open reading frame coding for the luciferase gene (Fluc) in a proprietary plasmid (Taconic-Artemis, Cologne, Germany). The CAG-LSL-*MYCN*-IRES-Fluc vector (LSL-*MYCN*) was introduced into the *ROSA26* locus of B6S6F1 embryonic stem cells by recombinase-mediated cassette exchange (Figure 1a). Recombinant clones were isolated, validated by Southern blotting and mice were generated by injection into tetraploid blastocysts. LSL-*MYCN* mice were crossbred with Dbh-iCre mice.^19^ Genotyping and confirmation of Cre-mediated recombination were performed as previously described.^15^ Primer sequences are provided in Supplementary Table 1. Abdominal tumors were detected by weekly palpation, and confirmed by high-frequency ultrasonography using a Vevo2010 device (Visualsonics, Toronto, ON, Canada) and/or by *in vivo* luciferase imaging.^15^ Time to tumor detection was displayed as tumor-free survival in Kaplan–Meier analysis. Growth curves for tumors were obtained by volume measurement using high-frequency ultrasonography.

### Gene expression analysis

The RNeasy Micro Kit (Qiagen, Hilden, Germany) was used to isolate total mRNA from cells or tissue, and cDNA was generated by SuperScript II Reverse Transcriptase (Invitrogen, Darmstadt, Germany). qPCR was performed using the TaqMan Fast Advanced Master Mix (Applied Biosystems, Darmstadt, Germany) and the StepOnePlus Real-Time PCR System (Applied Biosystems) according to the manufacturer's instructions. *Cad1*, *MYCN*, *Dbh*, *Th* and *Phox2b* mRNA expression levels were normalized to endogenous *Gapdh* and calculated using the dd-Ct method using Biogazelle software (Biogazelle, Ghent, Belgium) as previously described.^29^

### Western blot analysis

Cells or small tissue slices were lysed on ice in RIPA buffer (50 mM HEPES, 10 mM NaCl_2_, 1% NP-40, 0.1% SDS and 1% Triton X-100) supplemented with cOmplete Protease Inhibitor Cocktail Tablets and PhosSTOP Phosphatase Inhibitor Cocktail Tablets (Roche, Mannheim, Germany) and 30 μg of the resulting proteins were separated on 10% SDS-PAGE then transferred to Amersham Hybond-C Extra (GE Healthcare, Solingen, Germany) membranes. Membranes were blocked in 5% milk powder in TBS-T_0.1_ then incubated with primary antibodies against Brd4 (1:200; #sc48772; Santa Cruz, Heidelberg, Germany), E2f1 (1:1000; #AF4825; R&D Systems, Minneapolis, MN, USA) or MYCN (1:1000; #9405; Cell Signaling, Frankfurt am Main, Germany) and Actin (1:2,000; #A3853; Sigma-Aldrich, Taufkirchen, Germany) or Gapdh (1:2000; #MAB374; Millipore, Darmstadt, Germany) as a loading control. After washing twice with TBS-T_0.1_, membranes were incubated 1h at room temperature with horseradish peroxidase-conjugated secondary antibodies against mouse IgG (1:2000; #NA9310V; GE Healthcare), rabbit-IgG (1:2000; #NA9340V; GE Healthcare) or sheep IgG (1:2000; #HAF016; R&D Systems). Protein detection and visualization were performed as described previously.^30^

### Immunhistochemistry and electron microscopy

Briefly, 3-μm-thick sections of formalin-fixed paraffin-embedded tumors and adrenals were deparaffinized, and antigen retrieval was performed by boiling the section in citrate buffer at pH 6 or EDTA at pH 9 for 20 min. Staining was performed as previously described^31^ using anti-cleaved caspase 3 (#9661, Cell Signaling, 1:200), anti-tyrosine hydroxylase (ab76442; Abcam; 1:200), anti-Ki-67/Mib-1 (RM-9106, Dako Deutschland GmbH, Hamburg, Germany, 1:25) and anti-Ncam1 (ab6123; Abcam, Cambridge, UK; 1:500) as primary antibodies. Corresponding secondary antibody detection kits for reduced background in murine tissues were used (Histofine Simple Stain Mouse MAX PO, Medac, Hamburg, Germany), and antibody complexes were visualized using an automated stainer (LabVision Autostainer 480S, Thermo Scientific, Langenselbold, Germany). All slides were scanned with a Pannoramic 250 slide scanner (3D Histech.com Budapest, Hungary, Electron microscopy was performed as previously described.^32^

### Array comparative genome hybridization (arrayCGH)

DNA was isolated using the DNeasy Blood & Tissue Kit (Qiagen) according to the manufacturer's instructions. ArrayCGH was performed using a 180K (AMADID 027411) mouse whole-genome arrays (*n*=13; Agilent Technologies Santa Clara, CA, USA. Random primed labeling (BioPrime ArrayCGH Genomic Labeling System, Invitrogen) was used to label 400 ng of tumor DNA and matched control DNA with Cy3 and Cy5 dyes (Perkin Elmer, Waltham, MA, USA, respectively. Hybridization and washing were performed according to the manufacturer's instructions (Agilent Technologies). Fluorescence intensities were measured on an Agilent scanner (G2505C, Agilent Technologies). Data were extracted using the Feature Extraction v10.1.1.1 software program (Agilent Technologies), and further processed with arrayCGHbase (http://medgen.ugent.be/arraycghbase). Gains and losses were determined using the circular binary segmentation algorithm.^33,34^

### mRNA expression profiling

Primary murine tumors, normal murine adrenals and treated mNB-A1 cells were profiled on Affymetrix Murine 430 version 2 oligonucleotide microarrays according to the manufacturer's protocol. Microarray profiling results for eight LSL-*MYCN*;Dbh-iCre tumors have been deposited at the Gene Expression Omnibus under accession number GSE51297. Microarray profiling results for mNB-A1 cells have been deposited at Gene Expression Omnibus under accession number GSE57810. Profiles for three non-malignant adrenals from wild-type mice were used as controls. These profiles have been previously described by Molenaar *et al.*^22^ Profiles were compared with mRNA profiles of tumors from ALK-transgenic and TH-MYCN mice that have been published previously.^15^ Microarray.CEL files were normalized and summarized to gene levels using the Bioconductor repository of the R statistical language to do gcRMA normalization.^35^ Probes with a log2 expression of <5 in <11 of the 14 samples were considered not expressed and filtered out. Only the probe with the highest average expression over all samples was retained for each gene.

### miRNA expression profiling

Murine mature miRNA expression levels were quantified using the stem-loop reverse transcription-qPCR platform (Life Technology, Darmstadt, Germany). Briefly, 60 ng of total RNA was reverse transcribed using the rodent stem-loop RT Megaplex primer pools A and B (v2.0) followed by a 12-cycle pre-amplification according to the manufacturer's instructions. Pre-amplified cDNA was diluted and quantified using miRNA-specific Taqman assays (Life Technology) in a 3.5-μl qPCR reaction containing 1.5 μl of Taqman assay (1/17 dilution of 20X solution), 1.75 μl Taqman gene expression master mix, 0.02 μl of cDNA and 0.23 μl of water on a 7900 HT qPCR system (Life Technology). Raw miRNA expression values were filtered using a Cq-cutoff of 32, and normalized using the global mean, as previously described.^36,37^

### GSEA

The GSEA software was used to identify pathways or groups of functionally related genes deregulated in tumors from LSL-*MYCN*;Dbh-iCre mice compared with normal adrenal gland.^11,38^ GSEA was run on the collections of 3272 curated gene sets (c2) from version 3.1 of the MSigDB.^39^ Gene sets with less than 15 genes or more than 500 genes were excluded from the analysis. Gene sets with a false discovery rate ⩽0.25 and a nominal *PP*⩽0.05 were considered significant. The gene ranking metric in the weighted enrichment score was the two-sided signal-to-noise ratio, and *P*-values were calculated using 1000 permutations of the phenotype.

### Gene signature scores

Gene signature scores were calculated with adaptation of a previously reported algorithm.^40^ The LSL-*MYCN*;Dbh-iCre signature was composed of the top 100 most differentially up- and downregulated genes in LSL-*MYCN*;Dbh-iCre tumors compared with normal adrenal gland. The signature score was calculated for a panel of 967 cancer cell lines in the Cancer Cell Line Encyclopaedia,^13^ for which normalized gene expression data were downloaded from http://www.broadinstitute.org/ccle/home. Signature scores based on the expression values of MYC-regulated mRNAs or miRNAs, as previously defined by Westermann *et al.* and Mestdagh *et al.*, respectively,^14,41^ were calculated for a series of tumors from ALK^F1174L^,^15^ LSL-*MYCN*;Dbh-iCre mice and normal adrenal gland tissue. Additional MYC(N) signatures, retrieved from curated gene sets (c2) from version 3.1 of the MSigDB,^39^ were calculated for mNB-A1 cells treated with either JQ1, MLN8237 or DMSO. The published PUISSANT_NB_JQ1 signature^22^, composed of the 316 genes differentially expressed in neuroblastoma cell lines upon JQ1 treatment, was calculated for mNB-A1 cells treated with JQ1, MLN8237 or DMSO.

### Human neuroblastoma signature

A human neuroblastoma signature was generated using a published data set of 69 human primary neuroblastomas.^41^ The signature was composed of the most differentially expressed genes (Rank Product analysis, *P*<0.001) in high-risk compared with low-risk patients, either with or without inclusion of *MYCN*-amplified neuroblastomas, resulting in the human neuroblastoma signature and human non-*MYCN*-amplified neuroblastoma signature, respectively. To compare these gene signatures with expression data from LSL-*MYCN*;Dbh-iCre tumors, only genes with a known human and murine homolog were retained, yielding a list of 10 433 genes for further analysis.

### Establishing of a cell line from a LSL-*MYCN*;Dbh-iCre tumor

Murine tumor was minced manually with scissors and the pieces digested with 2 mg/ml collagenase in PBS for 30 min at 37 °C. Tumor pieces were passed through sieves with different pore sizes (400, 100, 70 μm) to obtain a cell suspension. Cells were maintained in RPMI medium supplemented with 10% fetal calf serum, penicillin (100 U/ml), streptomycin (100 μg/ml), 1% N2 and 2% B27. The mNB-A1 cell line was in continuous culture for more than 4 months, and all experiments performed here have been obtained after 3–4 months in culture. Cells were seeded onto 96-well plates and treated with 250 nM JQ1 (BPS Bioscience, San Diego, CA, USA) or MLN8237 (Axon Medchem, Groningen, The Netherlands). Metabolic activity was analyzed by 3-[4,5-dimethylthiazol-3-yl]-2,5-diphenyltetrazolium bromide (MTT) assay (Roche). We used this metabolic activity as a surrogate for the number of living cells, thus, cell viability.^42^

### Engraftment of mNB-A1 cells into nude mice

Six-week-old female athymic NCR (*nu/nu*) mice were subcutaneously inoculated in the left flank with 10^7^ cells derived from the LSL-*MYCN*;Dbh-iCre tumor suspended in 200 μl BD Matrigel (BectonDickinson, Heidelberg, Germany). Mice were measured for tumor growth three times per week using the formula (breadth · length · height)/2.

### JQ1 treatment of transplanted tumors from LSL-*MYCN*;Dbh-iCre mice

A Murine tumor was minced manually with scissors and the pieces were digested with 2 mg/ml collagenase in PBS for 30 min at 37 °C. Tumor pieces were passed through a sieve with 400 μm pore size to obtain a cell suspension. Cells were washed with PBS and suspended in 1.5 ml Matrigel (BD Bioscience, Heidelberg, Germany) for subcutaneous inoculation (200 μl per mouse) into the left flank of 6-week-old female athymic (nu/nu) mice. Mice were treated with JQ1 (50 mg per kg body weight) or vehicle control (12.5% DMSO in PBS) twice daily by intraperitoneal injection for 3 consecutive days when the volume of the subcutaneous tumor reached 500–1000 mm^3^, and animals were killed 4 h after the last injection. Half the tissue was snap-frozen in liquid nitrogen then stored at −80 °C and the other half was formalin fixed and paraffin embedded for immunohistochemical analyses.

### Statistical analysis

Statistical analyses were conducted using Graph Pad Prism 5.0 (GraphPad Software Inc, San Diego, CA, USA). Kaplan–Meier analyses were used to analyze overall survival. Mean relative apoptosis (calculated by positive staining for cleaved caspase 3) and proliferation (calculated by Ki-67 expression) in the transplanted LSL-*MYCN*;Dbh-iCre tumors from the control and JQ1-treated groups of mice was calculated from three representative images of each tumor using ImageJ 1.47 (National Institutes of Health, Bethesda, MD, USA). Significance was calculated by Student's *t*-test (*P<0.05, **P<0.01, ***P<0.001). Differential gene expression analyses were performed using the RankProd package in the R statistical language.

## Figures and Tables

**Table 1:** Genomic aberrations in tumors from LSL-*MYCN;*Dbh-iCre mice compared with tail

*LSL-MYCN; Dbh-iCre tumors*			*Human neuroblastoma*			
*Chromosomal region*	*Genomic aberration*	*Frequency (%)*	*Syntenic region*	*Genomic aberration*	*Frequency (%)*	*Reference*
1	Partial gain	8	2q	Gain	12	Vandesompele *et al.*^43^
1	Focal loss	8	1q41, 1q32.2, 1q32.3			
3	Gain	69	1q	Gain	Often	Schleiermacher *et al.*^10^
6	Gain	38	7	Gain	40	Stallings *et al.*^9^
6	Focal gain	15	3p25.3			
8	Focal loss	15	–			
11	Partial gain	31	17q	Gain	50	Vandesompele *et al.*^8^
12	Gain	23	7q	Gain	12	Stallings *et al.*^9^
16	Focal loss/gain	8	16q13.3			
16	Focal gain	8	21q22.13, 21q22.2, 21q22.3			
