# Supplementary figures and images for: A Cre-conditional MYCN-driven neuroblastoma mouse model as an improved tool for preclinical studies
Source: Oncogene. 2014 Sep 1;34(26):3357–68. doi: 10.1038/onc.2014.269 (PMC4487199; doi:10.1038/onc.2014.269)

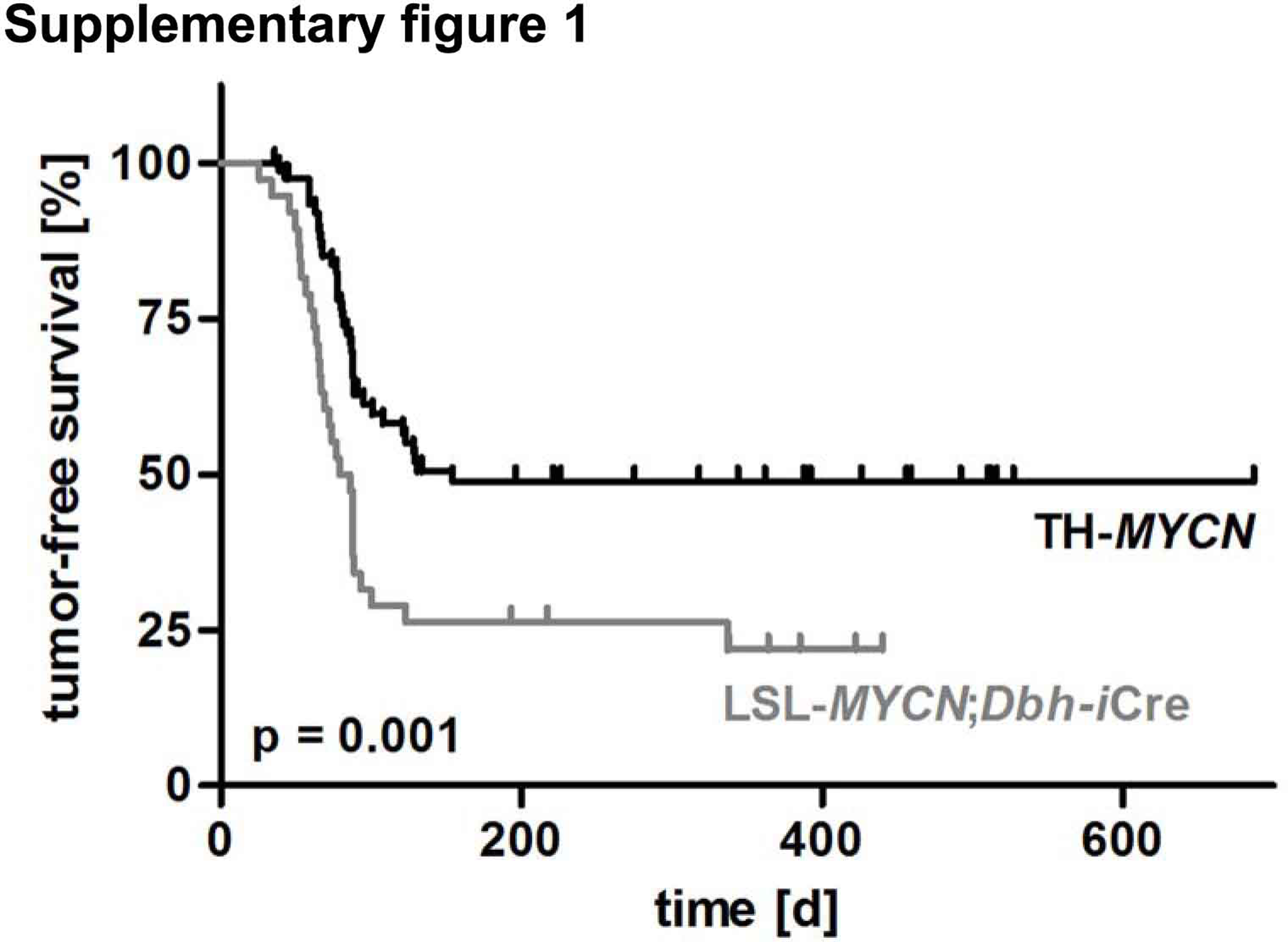

Supplement: Supplementary Figure 1 [file onc2014269x1.tif]

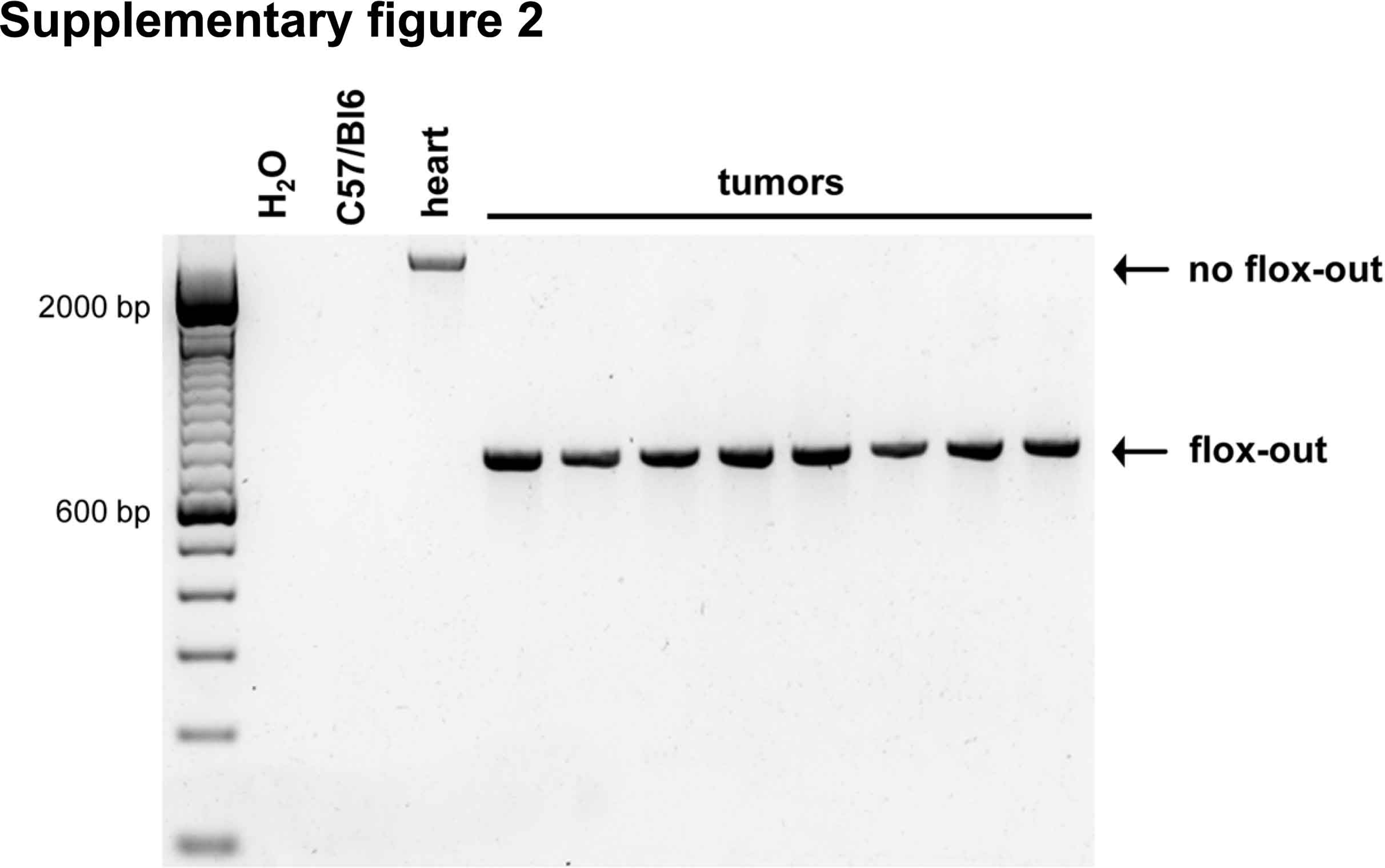

Supplement: Supplementary Figure 2 [file onc2014269x2.tif]

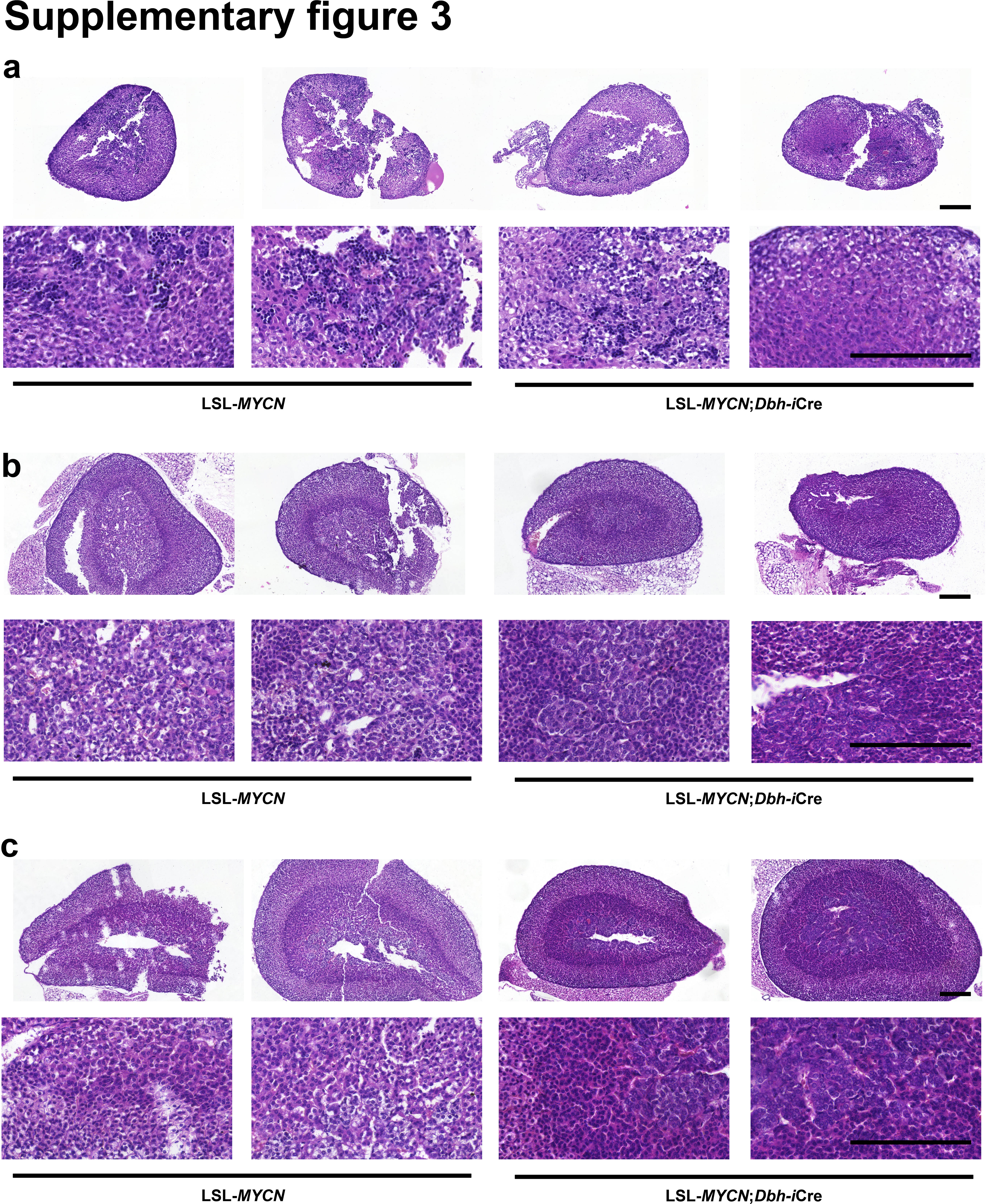

Supplement: Supplementary Figure 3 [file onc2014269x3.tif]

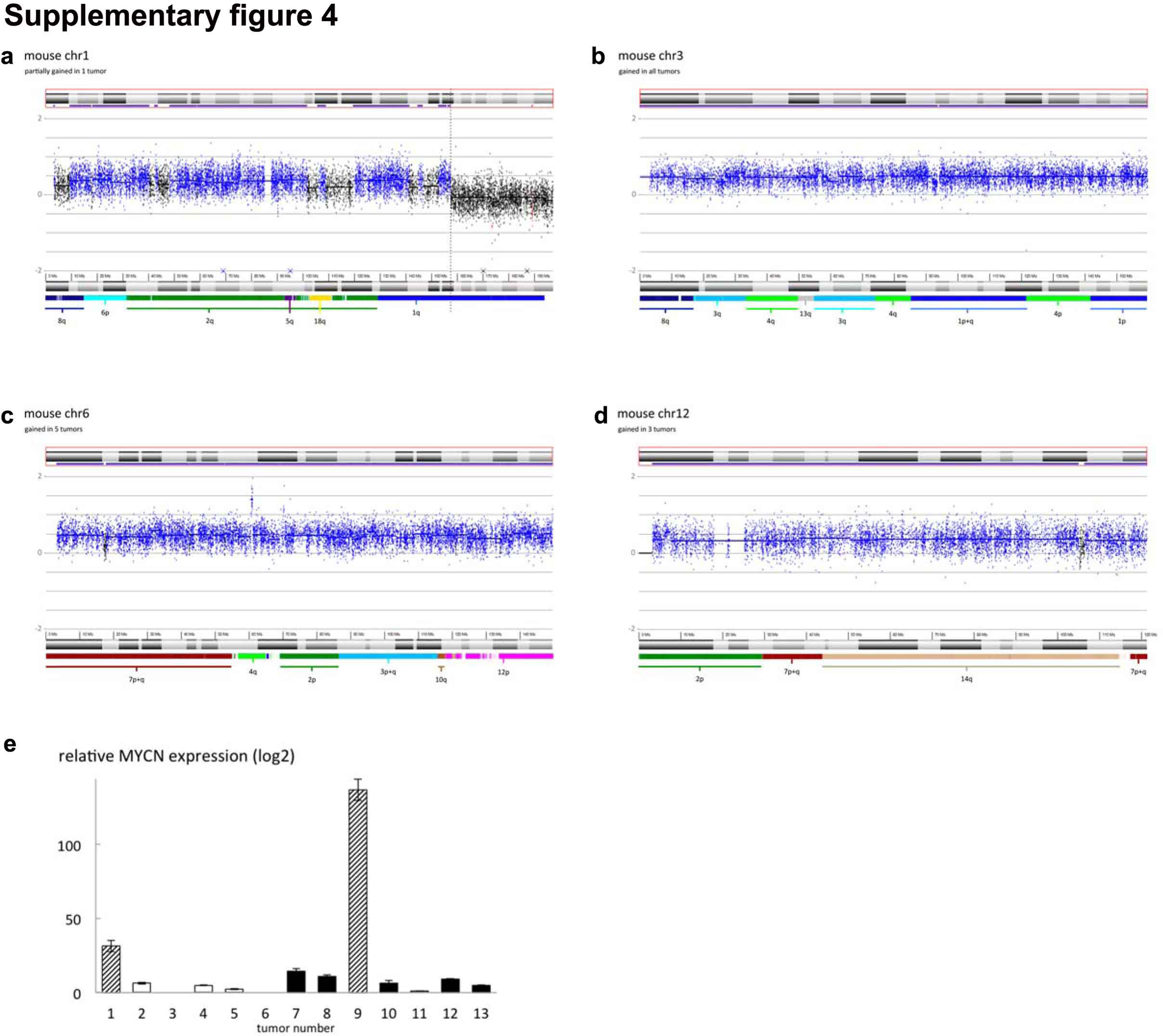

Supplement: Supplementary Figure 4 [file onc2014269x4.tif]

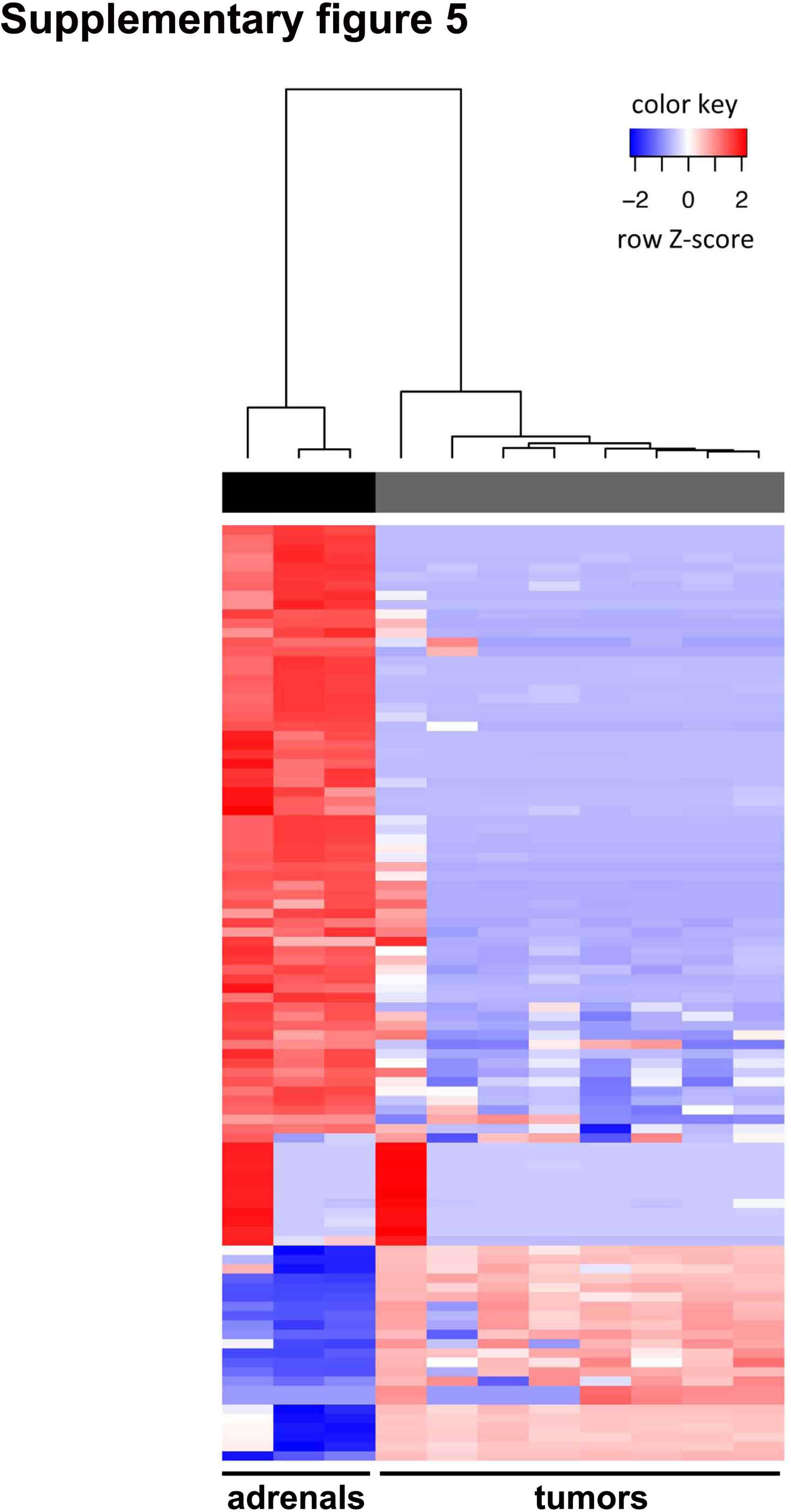

Supplement: Supplementary Figure 5 [file onc2014269x5.tif]

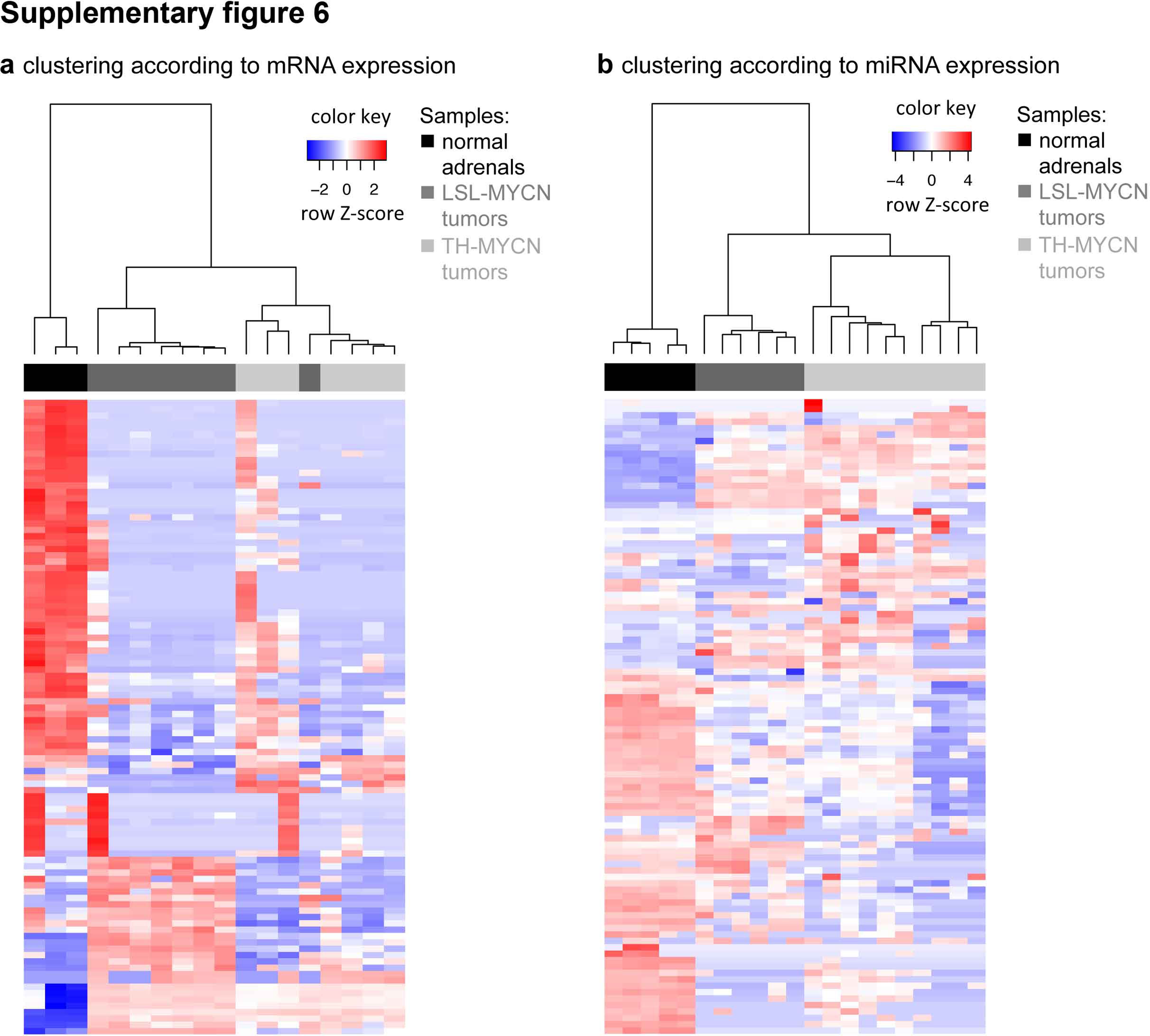

Supplement: Supplementary Figure 6 [file onc2014269x6.tif]

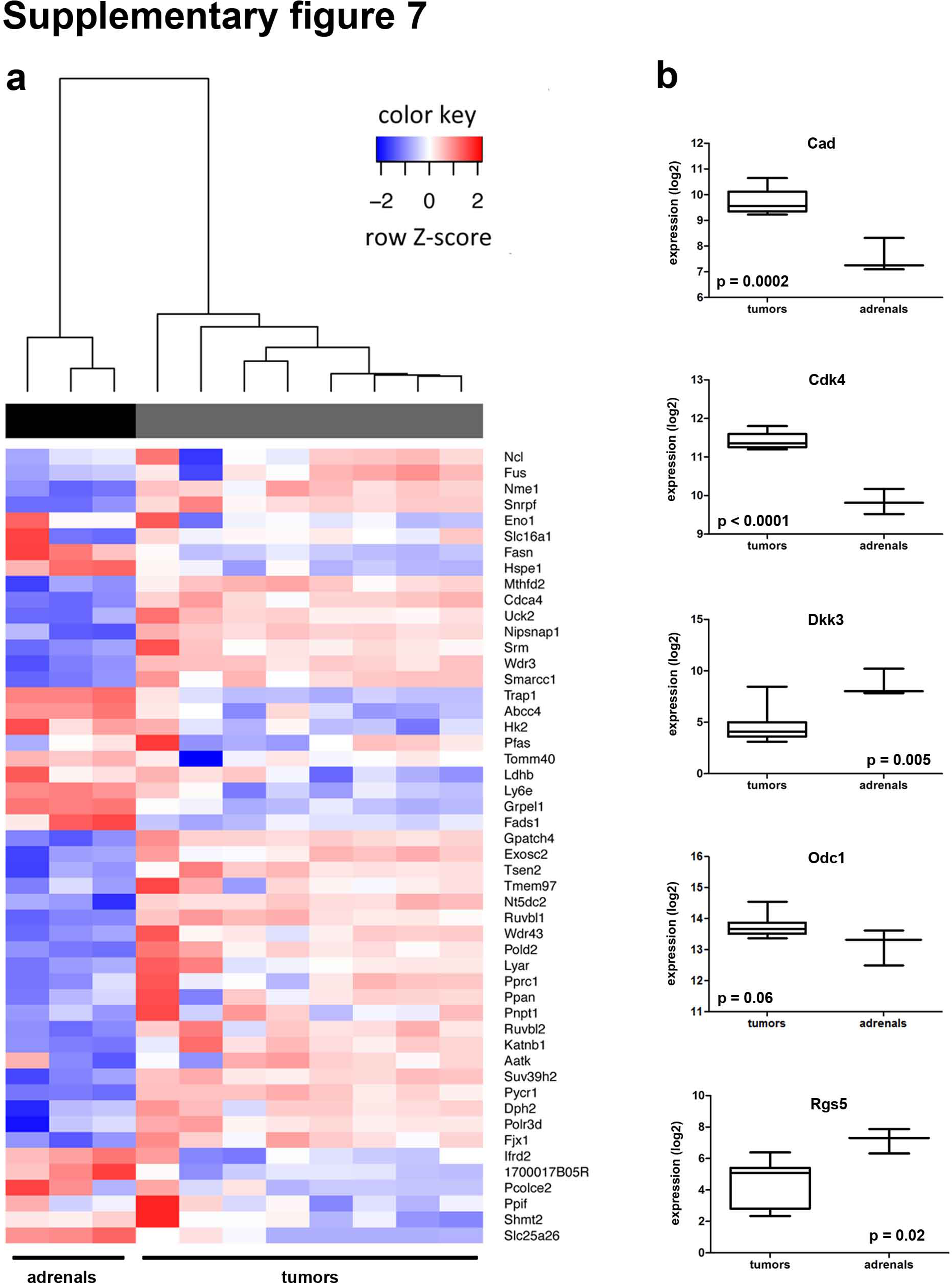

Supplement: Supplementary Figure 7 [file onc2014269x7.tif]

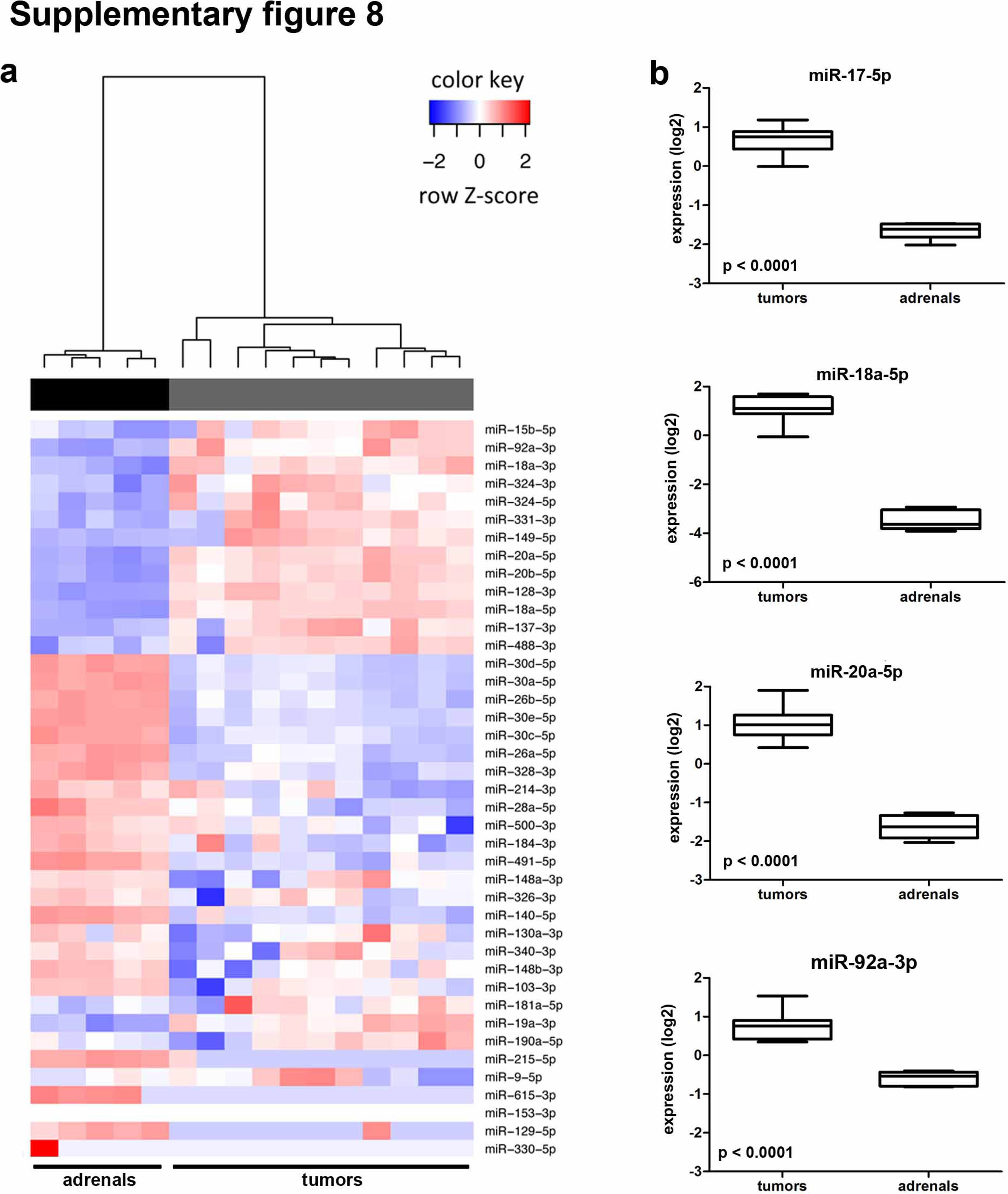

Supplement: Supplementary Figure 8 [file onc2014269x8.tif]

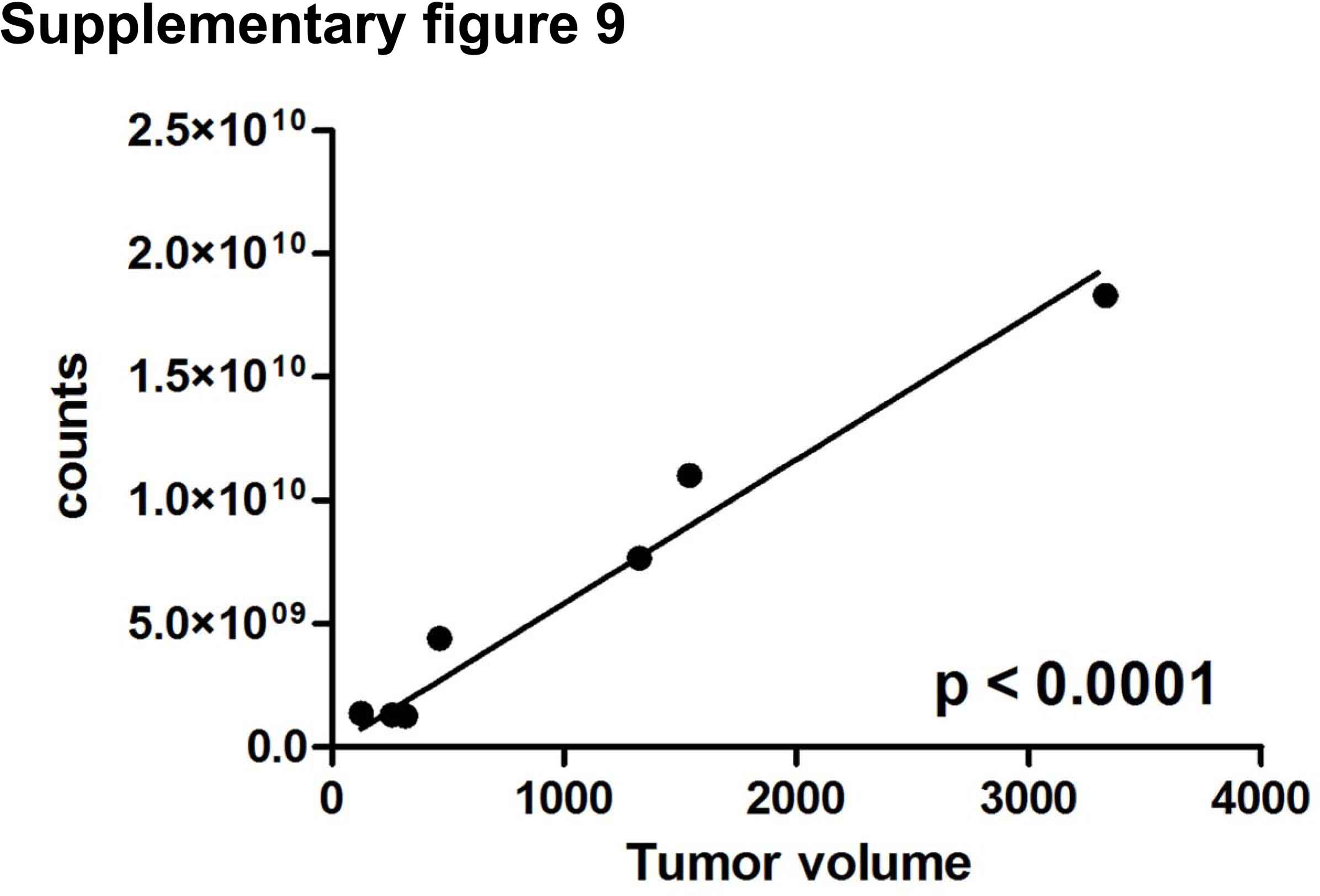

Supplement: Supplementary Figure 9 [file onc2014269x9.tif]

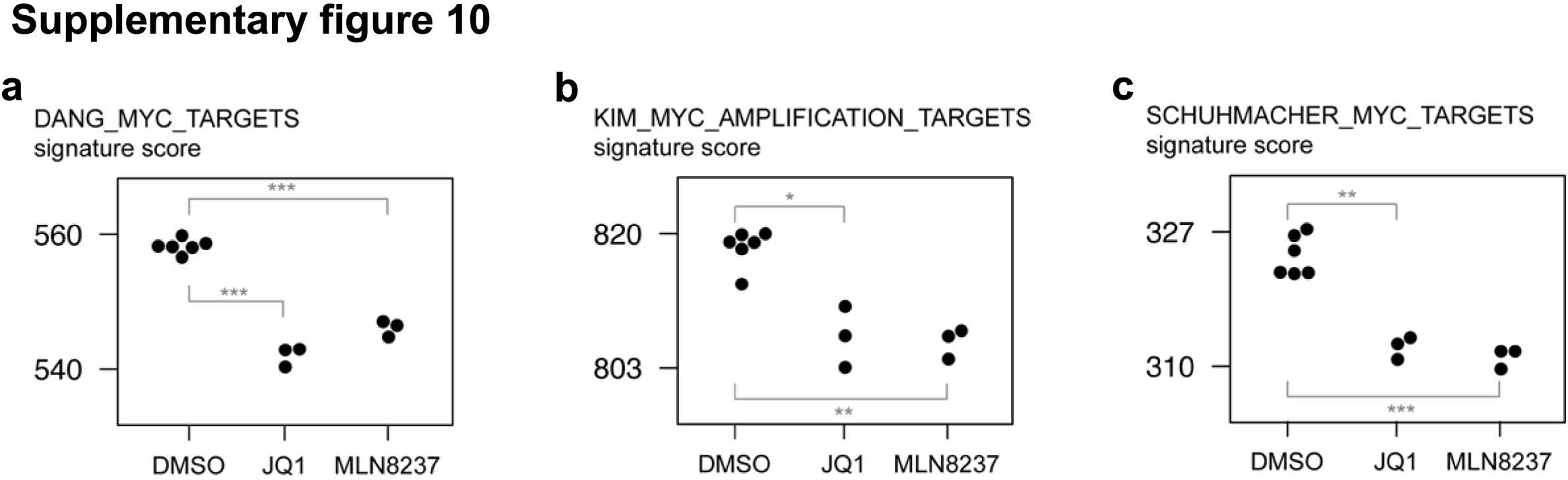

Supplement: Supplementary Figure 10 [file onc2014269x10.tif]

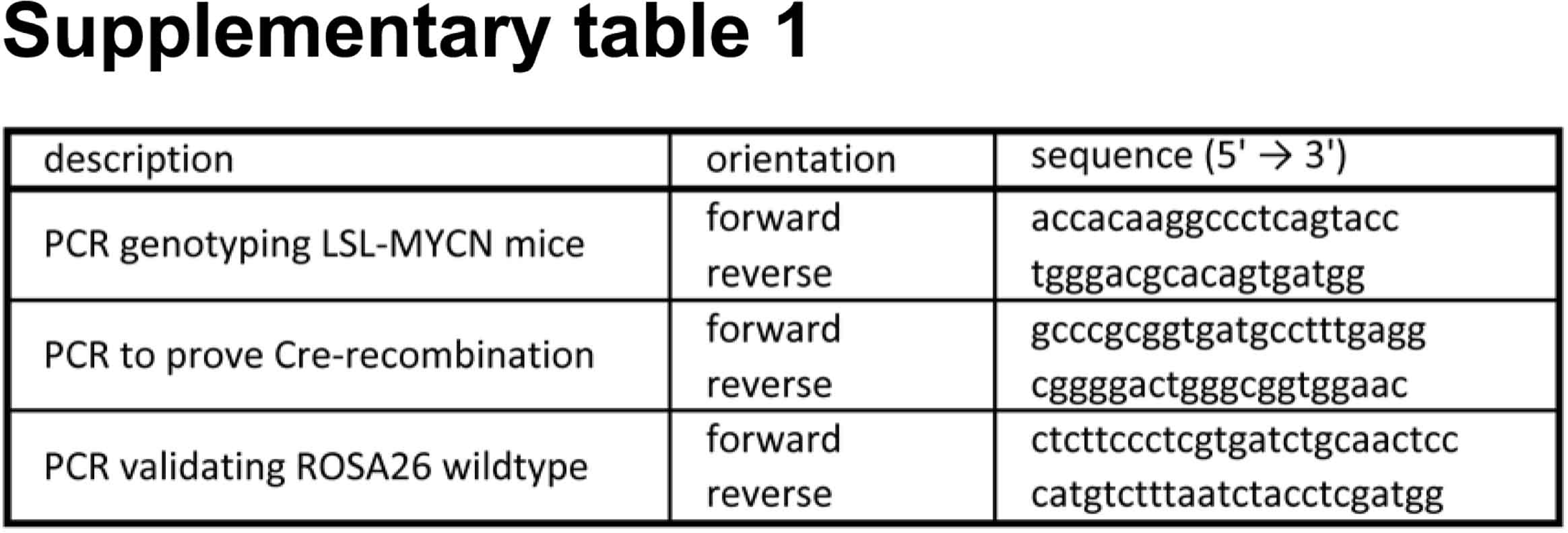

Supplement: Supplementary Table 1 [file onc2014269x11.tif]
